# Women’s perceptions of breast cancer risk and prevention: insights into knowledge gaps and lifestyle attitudes

**DOI:** 10.1186/s12889-026-27291-7

**Published:** 2026-04-10

**Authors:** Henrik Jonsson, Mariann Hedström, Åsa Grauman

**Affiliations:** 1 Region Uppsala, Eva Lagerwalls väg 1, Uppsala, SE-756 43 Sweden; 2 Department of Public Health and Caring Sciences, Uppsala University, Box 564, Uppsala, SE-751 22 Sweden; 3 Centre for Research Ethics and Bioethics, Uppsala University, Box 564, Uppsala, SE-751 22 Sweden

**Keywords:** Breast cancer, Risk, Perception, Health communication, General public, Prevention, Health promotion, Lifestyle, Illness representations, The Common-Sense Model of Self-Regulation

## Abstract

**Background:**

Breast cancer (BC) is the most common cancer among women. Promoting healthy lifestyle behaviours at the population level has the potential to prevent a substantial proportion of cases. Preventive interventions should be grounded in the target population’s existing beliefs and perceptions. Therefore, this study aimed to explore women’s perceptions of BC, their attitudes toward engaging in preventive behaviours, and their needs for risk information.

**Methods:**

Semi-structured individual interviews were conducted during spring 2025, with 16 Swedish women, aged 24–68. The interview guide was informed by The Common-Sense Model of Self-Regulation. The data were inductively analysed using thematic analysis.

**Results:**

Four themes were developed. (I) *Experience and representation shape women´s emotional and cognitive preunderstanding*: Women had clear perceptions of the consequences of BC but were largely uninformed about BC causes. They struggled to envision how lifestyle factors could influence BC risk, due to knowledge gaps about underlying mechanisms and because it did not correspond with the affected women they knew. (II) *Self-perceived risk: a mix of feelings and compensating reasoning*: Women had difficulties assessing their own risk due to uncertainty about whether their lifestyle was healthy enough and some acknowledged that their logical reasoning was overshadowed by their emotions. (III) *Willingness to change lifestyle: it is more than reduced risk of breast cancer*: Efforts to pursue a healthy lifestyle were mostly guided by its impacts on present wellbeing. (IV) *Constructing uplifting messages that reach and teach without blame*: Current information in society was perceived as too general. The women therefore requested more actionable and uplifting messages educating them about why something poses a risk factor.

**Conclusion:**

The study found that women primarily learned about the impact of BC on patients’ lives through media stories, which often evoked fear and sadness. Perceived preventive actions were largely limited to early detection through self-examination and participation in screening programs. However, there were knowledge gaps regarding BC causes that current information in society fails to address. To guide women’s health decisions, risk communication should prioritize evidence-based explanations of how risk factors influence BC risk, rather than relying primarily on personal narratives.

**Supplementary Information:**

The online version contains supplementary material available at 10.1186/s12889-026-27291-7.

## Background

Breast cancer (BC) is the most common cancer among women globally [1, 2]. One in eleven women in Europe develops BC before the age of 75 [3]. However, the risk varies between women due to the multifactorial aetiology of BC, including age, genetic predispositions, density of the breast, environmental factors and exposure to female hormones. The exposure to female hormones depends on maternal age of first child, number of births, breast feeding, time of menarche and menopause, and hormone-replacement therapy [1].

Lifestyle factors such as insufficient physical activity, alcohol consumption and excessive weight after menopause also influence the BC risk [1], partly due to their impact on oestrogen levels and inflammation [4–6], and the carcinogenic compound in alcohol [6]. These three risk factors have been linked to about 30% of all post-menopausal BC cases in the US [7] and Italy [8]. About 74% of the adult Swedish population consumes alcohol [9], approximately 50% have overweight or obesity [10], and 36% do not reach recommendations for physical activity [11]. The increasing incidence of breast cancer (BC) attributable to lifestyle factors highlights the need for cancer prevention strategies that place greater emphasis on modifiable behaviours [12].

Risk perception is one aspect explaining health behaviour [13, 14], and can therefore guide the development of preventive interventions and health communication [15]. High perceived risk has for instance predicted participating in mammogram screening [16] and acceptance of preventive treatment [17, 18]. Meanwhile, risk perception involves more than the probability of being affected. The Common-Sense Model of Self-Regulation (CSM) is a theoretical framework that explains how individuals react to health threats based on their cognitive and emotional representations of the illness [15]. The cognitive representation of an illness includes perceptions of symptoms of the illness, its consequences on different life domains, the timeline of the illness, causal beliefs, personal control, treatment control and coherence of the illness [19]. Emotional representations involve negative emotional responses (concern, fear, anger, and distress) [20]. Holding negative emotional representations and high concern about BC are associated with a higher personal BC risk perception [21].

Illness representations are influenced by personal experiences of the illness, social comparison and images of stereotypical cases, as well as through communication with close ones, health care providers, and mass media [15]. Perceived similarity to the person who develops BC has proven to be the strongest predictor of perceived BC risk, followed by perceived BC prevalence and being close to a friend with BC [15] or having a relative with BC [16, 18, 22].

Studies have shown that women tend to overestimate the risk of developing BC [23], report high levels of concern about BC [24], and are more concerned about BC than about cardiovascular disease, even though the latter poses a higher actual risk [25]. One plausible explanation is that the national BC screening programme has likely influenced on BC awareness. Another possible explanation is that BC has been overrepresented in media coverage [26], often portrayed through sensationalist stories featuring stereotypical images of cancer patients and narratives emphasizing the negative impact of BC on various aspects of life [27]. Previous research also indicates that atypical cases, such as younger women under the age of 40, are overrepresented in media coverage [28]. Exposure to sensationalist information has been shown to increase the likelihood of negative affect, fatalism, higher perceived BC risk, and the perceived younger age of onset [27].

Other studies instead found that high-risk women tend to underestimate their BC risk [29, 30]. This tendency might reflect women’s knowledge of BC risk factors. Women have reported insufficient knowledge of BC risk factors and some women have expressed uncertainty about whether it is even possible to reduce their BC risk [25, 31, 32]. While the awareness of family history as a BC risk factor was high [29, 32–34], awareness of overweight and alcohol consumption as BC risk factors was much lower [29, 31, 34–36]. A study assessing knowledge of the link between alcohol and cancer in 14 European countries, including Sweden, showed high awareness of the causal role of alcohol for liver disease (90%), while only 53% for cancer in general and 15% for BC specifically [35]. Meanwhile, there is a common belief that stress and worry cause BC, although the evidence for these associations is limited [37–39]. Awareness of BC risk factors varies due to socioeconomic factors, reflecting knowledge inequalities [34, 35, 37]. Furthermore, patients with BC [40] and high-risk individuals [41] have perceived contemporary information about BC risk factors as moralizing and insufficient to enable informed choices.

Hence, future communication needs to be carefully considered and built on a deeper understanding of women’s perceptions of BC, existing knowledge gaps, as well as potential barriers and facilitators to lifestyle changes [13]. Therefore, this study aimed to explore Swedish women’s perceptions of BC, their attitudes toward engaging in preventive actions, and their needs for risk information.

## Methods

The study was an explorative qualitative interview study. The study was reported in accordance with the Reflexive Thematic Analysis Reporting Guidelines (RTARG) [42], which offer a framework that aligns with the interpretive, flexible, and reflexive foundations underpinning the Reflexive Thematic Analysis used in this study.

### Participants

Inclusion criteria were being a woman, aged 18 or older, and able to read and speak Swedish. The exclusion criteria were having a history of cancer and training in medicine or nursing, since this study aimed to explore a lay perspective.

Participants were recruited using various strategies to obtain a sample that varied in age, education, and family history of BC. Invitation to participate was spread through open posts on Facebook and Instagram, flyers put up at noticeboards at different campuses of Uppsala university and two workplaces, and through the network of researchers. Women who expressed their interest (but not were selected) to participate in another qualitative research study about colorectal cancer were invited to this study. That study had been advertised in a local daily newspaper. Most were willing to participate in this study but some declined due to their special interest in colorectal cancer. The age of individuals willing to participate was assessed to facilitate purposive recruitment and to ensure a diverse age distribution among the women. Participants were offered a gift certificate of 150SEK (≈ 13 €) as a token of appreciation. The final sample size (*n* = 16) was guided by the concept of information power [43]. The study had a broad aim to capture a wide range of experiences and perceptions of risk from a varied study population, indicating the need for a larger sample size. However, the high-quality dialogue reduced the number of participants needed. Participants’ characteristics are presented in Table 1.

**Table 1:** Characteristics of study sample, *n* = 16

Participant	Age	Education (completed)	Born in Sweden	Family history of breast cancer
1	35	Secondary school	Yes	Yes
2	68	University	Yes	No
3	50	University	Yes	Yes
4	28	University	Yes	Yes
5	48	Post-secondary education	Yes	Yes
6	46	University	Yes	No
7	26	Secondary school	No	No
8	57	University	Yes	Yes
9	66	University	Yes	Yes
10	31	Secondary school	Yes	No
11	55	Secondary school	Yes	No
12	64	University	Yes	No
13	24	Secondary school	Yes	Yes
14	24	University	Yes	No
15	56	University	Yes	No
16	52	Secondary school	Yes	Yes

### Data collection

The individual interviews were conducted in Swedish between March and May 2025 by HJ and ÅG using a semi-structured interview guide (Supplementary file). The interview guide was informed by the dimensions of the CSM [15, 20] and included questions about perceived prevalence, consequences, treatment control, personal control, emotional response, and causes of BC. In addition, questions about self-perceived risk, mental images of the typical BC patient, willingness to change lifestyle and needs for information were included. After exploring participants’ perceived causes of breast cancer, the interviewer briefly mentioned research-identified modifiable risk factors to enable further exploration of participants’ beliefs and attitudes. The interview guide was tested in three pilot interviews before data collection was initiated. No changes were made to the interview guide.

All participants received written and oral information about the study and signed an informed consent form before the interviews. Thirteen interviews were conducted online (video call through Zoom), two face-to-face at the University, and one by telephone. At the beginning of each interview, participants were asked background questions. The interviews, which lasted between 29 and 58 min (mean 44 min), were audio-taped and transcribed verbatim.

### Data analysis

The data were analysed inductively using thematic analysis according to Braun and Clarke [44, 45]. It was chosen for its flexible approach that recognizes the researchers’ subjectivity as a valuable resource in the analysis process. Themes are not something that passively “emerge” from the data, but the outcome of the researchers’ active choices [45]. The analysis started with familiarisation with the data by actively reading the transcripts. In the next step, the transcripts were coded by ÅG, whereby the codes were sorted into potential themes. During this iterative process, themes were refined either by combining related themes or splitting them into more specific ones, while continuously going back and forth between transcripts, codes, and themes. The final set of themes and subthemes was labelled with a brief description to capture their essence [44]. HJ and MH contributed to the analysis through interpretive discussions aimed at producing a deeper and richer analysis, rather than pursuing coding consensus, inter‑coder reliability, or the resolution of discrepancies.

### Reflexivity statement

HJ, a male MSc student and registered nurse with experience in oncology care, conducted most of the interviews. We reflected on how his gender, age (29 years old), and junior academic status might have shaped the interview dynamics. While a male interviewer could potentially inhibit discussion of sensitive experiences, his junior position and nursing background may also have put participants at ease. Despite not being asked directly, several women spoke openly about issues related to female identity, sexuality, and reproductive concerns, and we did not observe any systematic differences between interviews conducted by male and female interviewers. ÅG and MH, both middle‑aged women within the BC screening age span, recognised that their academic roles and pre‑understandings of risk perception and caring science could constitute barriers to fully accessing a lay perspective. ÅG, an associate professor of public health, led the analysis, which was shaped by her interpretive and theoretically informed lens with an ethical orientation.

## Results

**Fig. 1:**
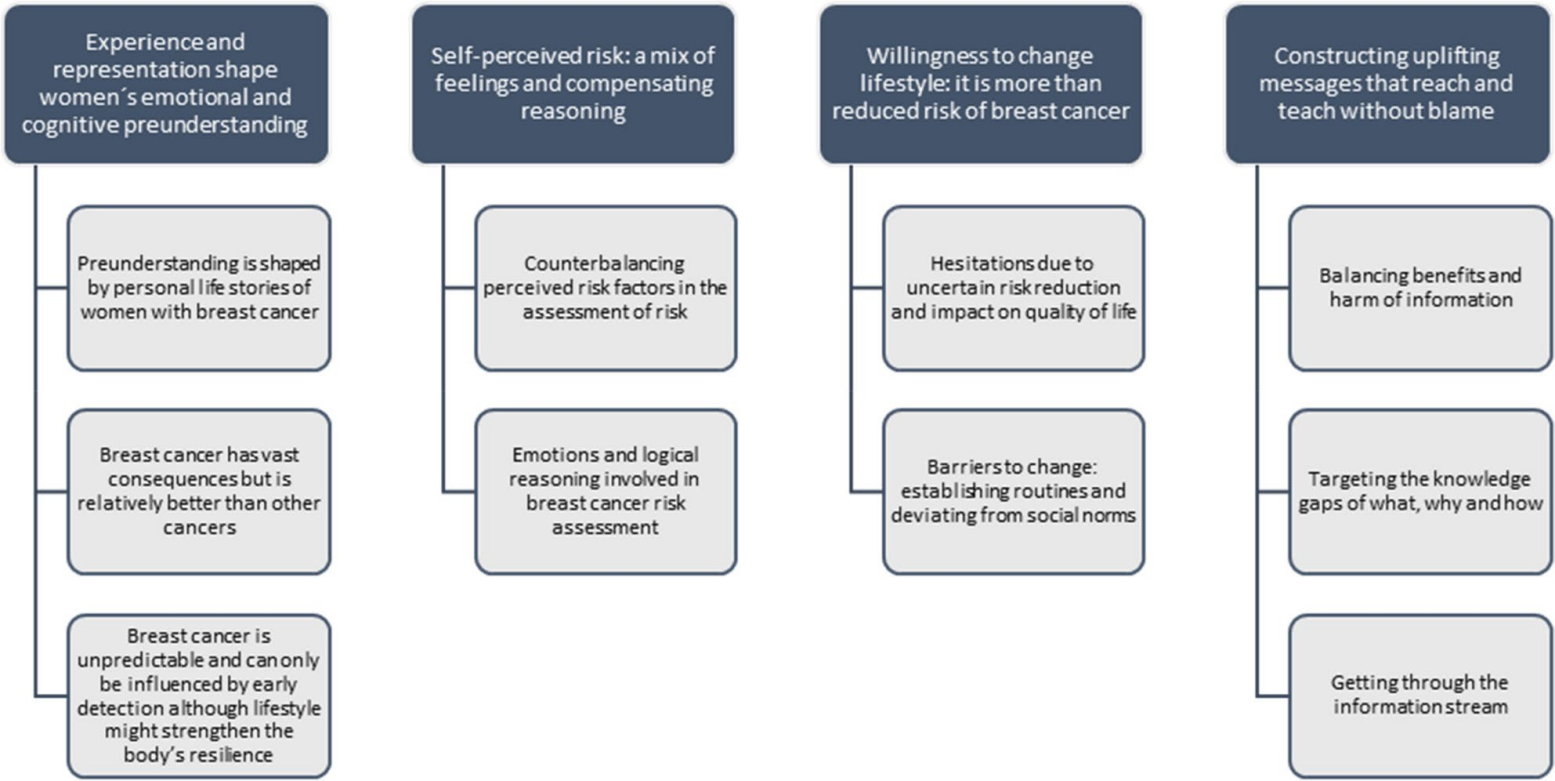
Themes and sub-themes

Four themes with ten sub-themes were developed (Fig. 1).

### Theme 1: Experience and representation shape women´s emotional and cognitive preunderstanding

This theme describes how women’s perceptions are shaped by personal stories leading to clear ideas about the consequences of BC and a perceived closeness through female identity, while lacking information about BC causes.

#### Preunderstanding is shaped by personal life stories of women with breast cancer

The women’s perceptions were largely shaped by personal connections to individuals affected by breast cancer, as well as by “Pink Ribbon” campaigns, articles in women’s magazines, and televised fundraising events. Knowing someone with breast cancer provided them with insight into its life-altering impact, often evoking strong emotions such as sadness and fear. Media coverage offered similar insights, often described as heart-breaking accounts of affected women’s lives. Campaigns also emphasized the importance of early detection, outlined symptoms, and encouraged self-examination. Meanwhile, the women noted that risk factors for BC were not brought up in these campaigns which one woman thought was “quite poor on their part.” Several women also acknowledged their limited understanding of disease mechanisms, saying they “didn’t really know what cancer is.” A few women stood out with a deeper pre-understanding, for example through professions such as microbiologist, subscriptions to newsletters from the Cancer Foundation, or proactively seeking information. Most women expressed that BC “feels” like a common illness among women, as it is frequently talked about and many knew several women who had been affected.

*“The reference I have is an old colleague who had it*,* and then there’s a lot of this… you know*,* there’s a lot in women’s magazines. They often have features about people who lived through BC*,* and a bit about how they look at life afterwards*,* and how one views breasts in general*,* identity in relation to one’s gender.”* (Participant 5).

Breast cancer has vast consequences but is relatively better than other cancers.

The participants perceived the prognosis as good, but pointed out that it depends on the type of BC and at what stage it is detected. Several women expressed that they “prefer breast cancer over other types of cancer,” as it is perceived as milder and has a better prognosis since the breast is an organ that can be surgically removed without compromising the body’s overall function.

*“I have an aunt who passed away from breast cancer and one who survived. So just based on that*, it’s fifty-fifty. My own research study *laughs* includes two people,* so there’s no evidence to support it. But I also know more women in my surroundings who are still alive.”* (Participant 1).

The women provided vivid descriptions of how BC affects an individual’s life—physically, psychologically, and existentially. They described the anxiety that arises when discovering a lump in the breast, the challenges of undergoing intensive treatments, the impact on daily life and relationships, and the long-term consequences. Several women spoke about how hair loss and undergoing mastectomy could lead to loss of femininity. One woman imagined feeling responsible for her husband and children if she became ill and could no longer be there for them in her role as wife and mother.

*“Breast cancer is a bit special because if you need to remove your breasts*,* it affects a woman deeply. You might have to think*,* ‘well*,* at least I’m alive*,*’ but it’s still hard to lose such sensitive parts of your body—parts that are connected to sexuality*,* but also to giving life. Maybe you’ve had children and breastfed them. I think that’s a real source of grief.”* (Participant 12).

#### Breast cancer is unpredictable and can only be influenced by early detection although lifestyle might strengthen the body’s resilience

Breast cancer was perceived as spontaneous mutations arising out of nowhere, due to bad luck or chance, although many were aware that BC can be hereditary. Many expressed that they “have no clue” what causes or protects against BC, and believed it could not be influenced personally, aside from self-examinations and BC screening to enable early detection. Some had heard that dense breasts carry a higher risk, and others wondered if larger breasts might be more susceptible due to having more tissue. A few women also mentioned smoking, hormones, radiation, chemicals and life traumas as possible causes. Many acknowledged that the risk increased with age, although one woman wondered whether the risk might actually decrease with age, as she had heard that BC screenings stop at a certain age.

*“I don’t think it’s anything specific*,* really. I imagine there isn’t a particular cause. Sometimes the body just mutates in a certain way. And you can’t really know where it comes from or why it affects that person.”* (Participant 13).

The participants stated that everyone is at risk of BC and had difficulties picturing the typical patient. The shared features that emerged were linked more to identity than to lifestyle as they intuitively imagined “an ordinary mother” around 40–50 years old. One participant highlighted Swedish ethnicity, reasoning that this image came from thinking about friends’ mothers who had been affected, and the type of women often portrayed in the media. One woman stated that “it’s not an overweight 55-year-old woman with alcohol problems.”

*“I picture a middle-aged woman quite clearly. Someone around 40 to 60 years old. She’s already had children*,* already has a career […] Just an ordinary lady. Or maybe just an ordinary mom. Mothers might be a common attribute I associate with BC.”* (Participant 14).

Many of the women expressed that they did not believe lifestyle influences the risk of BC, and that the “usual lifestyle advice” does not apply to BC. Participants referred to people they knew who had developed breast cancer, some had very healthy habits while others did not, reinforcing the idea that the disease strikes randomly.

*“I don’t think of BC as something you get from eating too much*,* not exercising properly*,* or sleeping too little. I think of it as hereditary. I could be completely wrong. I don’t believe you can… well*,* for example*,* if someone gets cancer in the liver or a kidney*,* maybe that’s because they’ve drunk too much alcohol or something like that. I don’t think of it as the result of an unhealthy lifestyle. I think it’s genetic—or just pure bad luck—that those cancer cells end up in someone’s breast.”* (Participant 6).

Lifestyle, although not perceived as a cause, was believed to strengthen the immune system. The general belief was that healthy habits “help against all diseases”. Healthy habits were described as avoiding stress, eating a balanced diet, exercising regularly, not smoking, getting enough sleep, and limited alcohol consumption. Regardless of their prior knowledge, participants expressed uncertainty about what levels of alcohol, exercise, or overweight that constitute a risk. Several participants thought it was interesting and unexpected to learn about modifiable risk factors during the interview, and asked questions about why and how these risk factors could cause BC, but had difficulty imagining the biological processes involved. Others reacted with scepticism, pointing to people they knew who didn’t fit the pattern.

### Theme 2: Self-perceived risk: a mix of feelings and compensating reasoning

This theme describes how women reason around their personal BC risk and how uncertainty, emotional responses and compensatory reasoning influence their risk assessment.

Counterbalancing perceived risk factors in the assessment of risk.

Women often assessed their BC risk through what we refer to as “compensating reasoning.” In this process perceived risk factors were balanced against the absence of other risk factors or presence of protective factors. Lifestyle and heredity were frequently treated as counterweights: a healthy lifestyle could be seen as offsetting a family history of BC, while the absence of heredity could downplay the impact of unhealthy habits. For instance, a woman with overweight perceived her BC risk as low because she felt she strongly resembled her mother, who never had BC.

*I do exercise quite a lot […] So I suppose I see it as chance playing a part. And I’m aware of my genetics*,* of course. Perhaps I should have a higher risk than others*,* I don’t know. But I don’t think so. I mean*,* logically I don’t think so*,* given my heredity. But emotionally*,* I believe it’s down to chance.* (Participant 9)

Several women expressed uncertainty about their BC risk, questioning whether they were “healthy enough.” Some participants also expressed uncertainty about how to evaluate the impact of their family history. For example, whether the BC within the family was caused by germline mutations or environmental exposure, or whether one affected family member was enough to constitute an increased risk. Some also believed that heredity mattered more if it came from the mother’s side. A few women with BC on one parent’s side still perceived their own risk as low, believing they had inherited genes from the other parent. Breast cancer risk was also devalued in the presence of other diseases that run in their family.

*“I think about the fact that my grandmother had it. So*,* I guess I have an increased risk. But this is probably due to lack of knowledge*,* because I don’t really know how it works. I believe she got it after working as a nurse and being exposed to radiation. And then I wonder*,* okay*,* if it affected her after she had children*,* could it still be part of my genetic makeup? But I don’t really know when it happened in relation to everything.”* (Participant 13).

#### Emotions and logical reasoning involved in risk assessment

Both emotions and rational reasoning shaped the participants’ personal risk assessment, and occasionally contradicted each other. A woman’s grandmother passed away from BC in her thirties, and therefore she felt her own risk decreased the more years that she lived beyond that age. She emphasized that she did not intellectually believe this, but emotionally it felt that way.

*“It feels like the older I get*,* the lower my risk of breast cancer becomes. And I don’t know if there’s any logical reasoning behind that*,* or if it’s just emotional—like the fact that my grandmother passed away when she was young. I feel like I’m in the safe hands of mammography*,* so to speak. And it feels like*,* no*,* my risk isn’t increasing. Rather the opposite. And that might go completely against what’s actually reasonable and logical. Maybe the risk increases with a woman’s age.”* (Participant 3).

Another woman also described a feeling of having a lower risk than others, even though she could identify certain risk factors in herself. She characterised this as a feeling that did not align with her rational understanding, explaining that she had always been healthy and that developing BC did not fit with her image of herself. Similarly, many women who perceived themselves to be at low risk still felt worried about being affected. One woman expressed that she thinks self-examination is important to enable early detection. Despite this, she avoided it because the thought of feeling a lump was too frightening, even though she simultaneously assessed her risk as low.

Most participants added that even if they perceived their own risk as low, they were still aware “as women” that they could be affected, since BC is such a common disease. Many had heard that everyone gets cancer if they live long enough, and for women, BC is among the cancers most likely to develop. Since BC is so closely associated with the female body, the feeling that it could happen to oneself increases. Being under surveillance due to high risk or participating in screening thus seems to contribute to a sense of security.

### Theme 3: Willingness to change lifestyle: it is more than reduced risk of breast cancer

Women required a strong effect on BC risk in order to change lifestyle. Instead, wellbeing was perceived as a stronger motivation for maintaining a healthy lifestyle. However, the women identified several barriers to succeeding with lifestyle changes.

#### Hesitations due to uncertain risk reduction and impact on quality of life

Some women expressed that they were willing to make any necessary changes to reduce their risk of BC. Others were reluctant to change unhealthy behaviours since they perceived their BC risk as low, already had a healthy lifestyle or were uncertain whether they needed to make changes. However, several women would be willing to make changes if they were told they belonged to a high-risk group or if they were diagnosed with BC. The women made trade-offs between reduced BC risk and the impact on quality of life when considering lifestyle changes. Some felt it was not worth changing a lifestyle that contributes to quality of life when it is uncertain whether it would protect them from BC. To be motivated to make changes, many needed a guarantee that they would not develop BC, or at least that research had shown a very strong correlation between lifestyle and BC.

*“Yeah*,* I guess I could consider it*,* but to be honest*,* it’s probably not something I’ll do. Mostly because I already feel like I have a daily life where I feel good. And yes*,* I do drink alcohol*,* absolutely. And I don’t really exercise much. But I think I do both at a level that works for me*,* where life feels enjoyable and my body feels fine. Could I change something? Well*,* I think if I knew I had a significantly increased risk of getting it*,* then yes*,* I could definitely consider making some changes. But I also think that not knowing whether I have an increased risk or not probably means I won’t think about it.”* (Participant 13).

On the other hand, a healthy lifestyle was seen as a way to achieve wellbeing in the present. The participants were overall more inclined to make small adjustments to their current lifestyle than complete lifestyle changes.

*I could probably do that*,* but I think I would actually do it more because I generally feel better from it*,* not primarily to reduce the risk of breast cancer*,* but that would come as a bonus. […]*

*(What would be the biggest motivator? ) That I feel so much better: it’s antidepressant*,* and then it would also help me reduce my BMI*,* I think. And in addition*,* it would help with what I have the greatest genetic risk for: cardiovascular disease.* (Participant 15)

Barriers to change: establishing new routines and deviating from social norms

#### Barriers to change: establishing new routines and deviating from social norms

Although many women perceived a need for change, they also identified numerous obstacles and some expressed low confidence in their ability to achieve it. In general, a stressful life situation was seen as a barrier. Obstacles to physical activity were difficulties establishing a routine and finding an activity one enjoys, as well as accessibility, costs, and physical disabilities.

Barriers to reducing alcohol consumption were more linked to social norms and culture. Younger women associated their drinking with student life, and one woman expected her consumption to automatically decrease as she got older and started working.

*“It’s the alcohol*,* I think*,* that might be the hardest. […] But alcohol is a social event*,* and sometimes that can be difficult. I think a lot about summers and things like that*,* when it can be harder. It works really well to have alcohol-free periods*,* but around Christmas*,* for example*,* it’s going to be… I know that it’s hard to stay alcohol-free then.”* (Participant 5).

One woman shared that she grew up in an “alcohol culture” and described having a biological craving for alcohol. However, she felt that understanding what happens in the body when drinking wine helped her refrain from alcohol.

### Theme 4: Constructing uplifting messages that reach and teach without blame

This theme captures the difficulty of conveying meaningful information to women amid an overwhelming abundance of health messages.

#### Balancing benefits and harm of information

Most women were positive towards receiving information about BC and its causes. It was considered important to spread this information to the public, as it encourages reflection on one’s lifestyle and supports making informed decisions. Some perceived the gained knowledge as an intrinsic value, even if it does not lead to any changes.

Yes, that’s interesting to know. But I don’t know if it would have changed much. I know that I am definitely in the risk group because I am a woman.

*Interviewer: “You are interested in learning about the information. But you feel that it might not make much difference. What is the value of the information?*

*“It lies in knowledge. Knowing*,* being able to pass it on.” (Participant 14)*.

Information about how BC can be prevented was perceived as reducing the feeling of BC as uncontrollable and the sense of powerlessness, while increasing a sense of hope. However, it was also perceived as discouraging, since it implies a personal responsibility that may lead to increased pressure and the feeling that one is doing wrong. One woman stated that shame is not a good starting point for change. Instead, information should feel supportive, coming from a place of genuine concern for the person, recognizing their worth and wishing them well.

*Well*,* of course I want to know how I can reduce the risk. But it’s also tough to carry that knowledge and then think*,* “Oh no*,* now I’m not doing this.” Will I get scared then? Like*,* okay*,* I ate two bags of chips tonight*,* but I wasn’t supposed to do that to reduce the risk. But now I did*,* did that increase the risk then? I don’t know… will knowing this also make me afraid?* (Participant 10)

Several also considered how such information might affect others. They could imagine that new information about how lifestyle influences BC risk might lead to patients feeling blamed, and that people might assume all patients have consumed a lot of alcohol. Therefore, it was considered important not to overemphasize personal responsibility.

*“But also*,* that it can be a little*,* how should I put it*,* a little dangerous too*,* because it can lead to people with breast cancer being blamed precisely because of the proven link: “Well*,* maybe it’s because you’ve been drinking a lot of alcohol*,*” even though you might not say it*,* but it can lead to this kind of silent shaming. Yes*,* or that you start to speculate*,* like*,* “could it have something to do with that?” or that the person themselves thinks*,* “oh no*,* will people start speculating about my alcohol consumption.”* (Participant 7).

#### Targeting the knowledge gaps of what, why and how

The women said that the most important aspect to include in information is what they themselves can do to reduce their risk. However, they felt that general information such as “it’s good to exercise and not drink alcohol” was insufficient and merely repeated what they already knew. Instead, they expressed a need to understand why something increases the risk, what happens in the body, and how much the risk is increased or reduced by modifying a risk factor.

*I want the latest scientific studies available. I want the hard facts… Why do we get it? How does it develop?* (Participant 5)

*I would rather have information about protective factors than risk factors. […] But then the important thing is: what can I do? What do I want to choose to do? And they’re somewhat connected. I mean*,* if drinking alcohol is a risk factor*,* then maybe I want the information to say: if you halve your alcohol consumption*,* your risk will decrease by this much.* (Participant 15)

One participant pointed out the importance of also informing about non-modifiable risk factors, so there is no misinterpretation that lifestyle is all that matters. One younger woman expressed that she didn’t think this type of information was relevant for people under 25.

The women were generally negative toward fear-based messaging without concrete advice. Instead, many called for positive, encouraging information about what reduces risk and the benefits of changing behaviour. The advice needed to feel achievable and close to their current lifestyle. Even if the message should be brief, the information should include links to further information and where to turn for support with lifestyle changes.

*Yeah*,* well*,* the research studies that exist and concrete levels*,* and that there are recommendations*,* sort of. […] Yes*,* highlight what is positive. Yes*,* but not scare tactics*,* rather the opposite. […] Like*,* improve your chances by making these small changes in your everyday life.* (Participant 1)

#### Getting through the information stream

The overwhelming presence of health-related messaging in society was seen as an obstacle to engaging with the information. The women felt they were exposed to an overload of cancer warnings, which gave the impression that “everything causes cancer.” The different messages were also perceived as contradictory, making it difficult to know what is true. Therefore, they believed that only the most certain and strongest associations should be communicated. Some felt most receptive when they actively sought out the information themselves. However, few had done so. Therefore, information needs to be easily accessible for those who look for it, but also proactively communicated to reach those who are not actively searching.

Most of the women expressed that BC screening was a suitable opportunity to provide information about BC risk factors, as they believed people are psychologically receptive at that time. Meanwhile, younger women suggested providing information at sexual health clinics. It was considered advantageous to receive information during personal contact. Beyond that, preferences for how to receive information varied widely. Many stated that the source of information was crucial, and many considered universities and the healthcare system as trustworthy sources.

*“I think it’s interesting when you search for that kind of information yourself. That’s because you’re genuinely interested in knowing the answers. But I haven’t done that either. Still*,* I’m interested in it. I think getting it through the media can be a good way. Getting it through social media or the news or something like that. Radio. It’s still stuff you take in. But it can also be quite a lot*,* because there’s a lot of talk about cancer in the media too. And then again*,* it becomes a question of what to trust.”* (Participant 14).

## Discussion

This study explored Swedish women’s, aged 24–68, perceptions of BC, their attitudes toward engaging in preventive actions and their need for information. The women perceived BC as spontaneous mutations arising out of nowhere and had difficulties imagining that lifestyle could influence BC risk, due to knowledge gaps about underlying mechanisms and because it did not correspond with the affected women they knew. They had difficulties assessing their own risk due to uncertainty about whether their lifestyle was healthy enough and some acknowledged that their logical reasoning was overshadowed by their emotions. Efforts to pursue a healthy lifestyle were mostly guided by its impacts on wellbeing in the present. Current information in society was perceived as too general. The women therefore requested more actionable and uplifting messages educating them about why something poses a risk factor.

As seen in previous studies [32, 46, 47], most women identified family history as a risk factor for BC, and referred to it when assessing their personal BC risk. While family history can increase the perceived BC risk [16, 18, 22], a lack of family history can contribute to a false sense of security as it has been shown to contribute to the underestimation of cardiovascular risk [48]. It was also common to believe that protective factors compensate for unhealthy ones. Knowing women affected by BC who maintained a healthy lifestyle led participants to question the influence of lifestyle on BC risk. Moreover, many attributed BC to luck or chance and expressed the view that ‘everything causes cancer,’ reflecting fatalistic perceptions of the disease as unpredictable and uncontrollable, consistent with previous research [46, 47, 49].

There was uncertainty about how to assess personal BC risk and how to apply risk factors to themselves. This included known factors such as family history. Beliefs that a family history on the mothers’ side poses a greater risk than on the father’s side have also been observed in an Australian study [50]. The participants also expressed that they perceived their lifestyle as fairly healthy but were unsure whether it was healthy enough. This relates to their uncertainty about the levels of physical activity, excess weight, and alcohol consumption that would increase risk. Comparable to previous studies [36], most participants underestimated the amount of alcohol consumption that poses a risk. Another Australian study found that although 70% of participants identified alcohol as a BC risk factor, only about 40% identified red wine as a risk factor. Furthermore, about one quarter of those who believed that alcohol increased BC risk still believed that red wine decreased risk [37]. This highlights that even when individuals are aware of a risk factor, considerable knowledge gaps may exist that hinder informed decision-making. A US study found that drinkers are more likely to believe that alcohol is not a risk factor for BC compared to those who did not drink [34]. They suggested that devaluation of the importance of their own risk factors might be a sign of high perceived personal control, and a reflection of socially accepted levels of consumption [34].

Although the median age of BC onset in Sweden is 66 [2], the women intuitively thought of the typical BC patient as a woman in her 40–50 s, which could be a results of overrepresentation of atypical BC patients in magazines [27]. One woman also concluded that the risk decreases with age based on the fact that BC screening is discontinued at age 75. Communicators should therefore strive to choose more age-diverse spokesmodels in BC campaigns.

In this study, as well as in previous research, women expressed an interest in information about BC and its risk factors [31, 51]. However, the participants in this study perceived current societal risk information as too general. To be informative, they requested information on why certain factors influence risk, including the biological mechanisms involved, the magnitude of the risk increase and the baseline level, as well as practical advice on how to reduce the risk. Several women also suggested that information on risk factors should be combined with guidance on symptoms to be attentive to, self-examination, and where to seek care if something is discovered. Presenting baseline risk levels is a recommended method for risk communication that has been shown to improve individuals’ understanding of risk and benefits [52]. Additional strategies include using plain language, visualizing risk using pictographs, clearly specifying the time frame (e.g., 5-year, 10-year or lifetime), and presenting risk in frequencies rather than percentages. It is also advised to present risk in absolute rather than relative terms, as relative risk, commonly used in media, can make changes appear larger than they are [52]. For example, instead of stating that lifetime BC risk is reduced by 15% if a woman maintains normal weight compared to obesity, it is clearer to state that the risk changes from 11.9% to 10.1%. Presenting BC risk alongside other competing health threats with higher risk (such as cardiovascular disease) might also provide nuance to the often overestimated perceived risk of BC among women [53].

Preferences for how risk should be communicated varied between the women in this study and those in previous research [31]. Breast cancer screening has been suggested as an appropriate context for information provision [31, 54, 55], as women are psychologically receptive to engaging with the information at that time [31], which participants in this study also stated. However, BC screening staff have also expressed concerns about how to talk about BC risk factors in a non-stigmatizing way, and expressed ambivalence about their role in health promotion [55]. Risk-stratified BC screening is currently being tested in several European countries. A qualitative study including women in France and the UK found that participants appreciated receiving their 5‑year breast cancer risk estimates. For women at high risk, the most important factors were specialist‑delivered, personalised risk communication that helped them feel reassured [56]. Younger women, who were not yet invited to BC screening, suggested receiving such information at the sexual health clinics instead. Some of the young participants expressed that lifestyle changes are not prioritized at their age, and information therefore was not relevant. This perceived irrelevance among younger women is problematic since the timeframe from menarche to first pregnancy represents a critical period when the breast tissue is particularly susceptible to carcinogens such as alcohol [57], and this calls for educational efforts targeted at younger women.

When considering lifestyle changes, the women made trade-offs between the impact on current quality of life and future BC risk. For many, the perceived reduction in BC risk was too uncertain to motivate change, and they expressed a need for stronger assurances of protection before altering their habits. Similar reasoning has been reported among Swedish cancer patients [40]. Fatalistic beliefs, such as “everything causes cancer” or “nothing can be done to prevent cancer,” may also foster a ‘live for the moment’ attitude, reducing concern for long-term health [49]. Several studies have found women unwilling to decrease their alcohol intake to reduce their cancer risk [58–60], regardless of their educational level or BC risk perception [58]. The reluctance to decrease alcohol consumption could reflect that alcohol has a strong social and cultural significance and that social life plays a strong part in the perceived quality of life [61]. On the other hand, the women felt that a healthy lifestyle contributed to present wellbeing, and considered wellbeing the main driving force for maintaining healthy habits. Previous research has also identified immediate outcomes related to wellbeing as key motivators for adopting healthy habits, such as looking more attractive, feeling happier [18], avoiding weight gain and hangovers [36], reducing menopause and depression symptoms, and reducing the risk of other medical conditions [18]. To encourage lifestyle changes, it may therefore be beneficial to also communicate these short-term outcomes when explaining how lifestyle can influence BC risk.

Many women acknowledged their need for lifestyle changes and had made numerous efforts throughout their lives They brought up barriers previously identified in research including a stressful life situation and costs [18]. The women requested support to enable lifestyle changes, such as making a plan, adapting physical activity to physical limitations, or receiving practical tips on reducing alcohol consumption. Managing weight loss without professional support can be especially difficult. Information about the correlation between overweight and BC, without offering support, can do more harm than good as it risks reinforcing negative feelings of failure and self-blame [41]. Furthermore, different social groups have different capacities to act on information, for example, an Australian study found structural limitations on working-class women’s possibility to act on health messages [47]. Likewise, beliefs about causation are closely connected to stigma, where the perception of personal control over risk factors increases blame on patients and reinforces the notion that the cancer is self‑inflicted [12, 40]. To prevent this, information about modifiable risk factors should be balanced with information about non-modifiable risk factors, and emphasise structural barriers to achieving a healthy lifestyle. Health promoters should be humble about these obstacles and aim to not only inform but to facilitate and support lifestyle changes for those who want to change.

### Strengths and limitations

The interview guide used in this study was informed by CSM, thus ensuring that essential aspects contributing to individuals’ representation of BC were covered, witch strengthens the credibility of the study [62]. That the interviews were performed by two of the authors could decrease the study’s dependability, as differences in interviewing style, probing strategies, and interactional dynamics could have produced some variation in how the interviews were conducted. To support dependability, both interviewers used the same semi‑structured interview guide and held regular discussions to align their approaches.

We strove to gather a variety of perceptions to increase credibility [62]. Since awareness of BC risk factors varies due to socioeconomic factors [34, 35, 37], women of various ages and educational levels were recruited through different recruitment strategies. We also reported the country of birth and family history of BC to further enable the reader to assess transferability to other settings. Almost all participants were born in Sweden and many expressed high trust in researchers and the health care system. The results may therefore not reflect the perceptions of women with primary school as the highest level of education, ethnic minorities or groups in society who hold mistrust against science and public authorities. However, the findings of this study are very similar to those of an Australian interview study [31], indicating that the perceptions and attitudes are not isolated to Sweden. The participants received a gift card as a token of appreciation, but as the amount was low, we do not believe the cards influenced study participation.

## Conclusions

The study found that women primarily learned about the impact of BC on patients’ lives through media stories, which often evoked fear and sadness. Perceived preventive actions were largely limited to early detection through self-examination and participation in screening programs. However, there were knowledge gaps regarding BC causes that current information in society fails to address. Also, in cases where the women were aware of certain risk factors, their understanding was not sufficient to apply the knowledge to themselves. To guide women’s health decisions, risk information should prioritize evidence-based details on how risk factors influence BC risk, rather than relying primarily on personal narratives. However, our findings suggest that information about the increased risk associated with individual factors may not be perceived as significant enough to motivate lifestyle changes on its own, presenting a challenge for promoting healthy behaviours among the public.

## Supplementary Information

Supplementary Material 1

## Data Availability

Due to the formulations of the informed consent form, study data cannot be made publicly available. Data are however available from the corresponding author upon reasonable request subject to ethical permissions and participant consent.
