# Supplementary material for: Women’s perceptions of breast cancer risk and prevention: insights into knowledge gaps and lifestyle attitudes
Source: BMC Public Health. 2026 Apr 10;26:1238. doi: 10.1186/s12889-026-27291-7 (PMC13085573; doi:10.1186/s12889-026-27291-7)
Supplement: Supplementary file 1 — Supplementary Material 1 [file 12889_2026_27291_MOESM1_ESM.pdf]

Supplementary file. Interview Guide – Public Perception of Personal Risk and Risk Factors for Breast Cancer.

**Interview guide was originally in Swedish*

Before interview: Ensure written informed consent is collected. Briefly introduce yourself. Repeat information about the study. Assess background variables.

Start audio recording.

Opening Questions, Identity

- What do you know about breast cancer from before?

Questions about illness perceptions of the breast cancer

Prevalence

- Do you have any idea how common breast cancer is in the population?

Consequences

- Do you have any idea how the disease affects the life of someone who is diagnosed?

Treatment Control

- What do you think about the possibility of treating or curing breast cancer?

Emotional response

- What feelings does the thought of breast cancer evoke in you?

Stereotypical Beliefs

- Do you have any idea who gets breast cancer? How do you imagine the typical patient?

Personal Control

- To what extent do you feel that you can influence your own risk of developing breast cancer in the future?

Causes

- What do you think causes breast cancer? *Can you think of several factors that might cause breast cancer?*

- Are there things you believe could protect against breast cancer?

- *You've mentioned several possible risk factors (such as…), which do you think has the greatest impact on risk?*

- *How important do you think lifestyle factors are compared to, for example, heredity, chance, or other exposures?*

- (You mentioned alcohol/overweight/physical inactivity as a risk factor). Research has shown that alcohol/overweight/physical inactivity increases the risk of breast cancer. What are your thoughts on that? How much alcohol/overweight/physical inactivity do you think one needs to drink before it becomes a risk for breast cancer?

Personal Risk Perception

- How do you view your own risk of developing breast cancer in the future?

- What causes do you think play the biggest role in your own risk of developing breast cancer in the future?

Attitudes Toward Lifestyle Changes

- Could you be willing to change something in your everyday life to reduce the risk of breast cancer? (e.g., alcohol consumption, exercise, weight loss)

- *Are there circumstances that would make such a change easier?*

- *Are there circumstances that could make such a change more difficult?*

- *Are there things you wouldn't consider changing in your everyday life?*

Risk Information

- Are you interested in receiving information about how you can influence your risk of breast cancer? *Is there anything specific you would like that information to include?*

- If so, how would you prefer that information about how lifestyle affects cancer risk to be communicated to you?

- Can you recall any occasion when you received information about what increases or decreases the risk of breast cancer? Can you tell me about that occasion? Where did you get that information? Do you remember how you reacted to it?

- Could information about how lifestyle affects cancer risk affect you negatively in any way?

Closing Questions

That was all the questions I had. [Provide a brief summary of what has been said]. Is there anything you would like to clarify? Is there anything we haven't talked about that you would like to add?

Thank you for your participation!

Follow-up Questions:

- Could you tell me more about that?

- What are your thoughts on that? Is it good or bad?

- What feelings does that evoke? How did it feel?

- Am I understanding you correctly that…
